# Omics Approaches in Hantavirus Research: Current Advances, Challenges, and Future Perspectives

**DOI:** 10.3390/biotech15030058

**Published:** 2026-07-23

**Authors:** Soroosh Najafi, Maryam Jojani, Kianoosh Najafi, Giovanni N. Roviello

**Affiliations:** 1 Scientific and Production Center “Armbiotechnology” NAS RA, 14 Gyurjyan Str., Yerevan 0056, Armenia; najafi1380@gmail.com; 2 School of Medicine and Surgery, University of Naples ‘Federico II’, Via S. Pansini 5, 80131 Naples, Italy; aidajojani@gmail.com (M.J.); najafi2725@gmail.com (K.N.); 3 Institute of Biostructures and Bioimaging (IBB), CNR National Research Council of Italy, 80145 Naples, Italy

**Keywords:** hantavirus, multi-omics, genomics, transcriptomics, proteomics, metabolomics, precision medicine, host–pathogen interaction, HFRS, HCPS, bioinformatics, therapeutic targets

## Abstract

Hantaviruses are zoonotic RNA viruses responsible for two severe human diseases: hemorrhagic fever with renal syndrome (HFRS) and hantavirus cardiopulmonary syndrome (HCPS). Worldwide case fatality rates vary from 1% to 40%. Although single-layer genomics, transcriptomics, proteomics, and metabolomics studies have advanced our understanding of hantavirus biology, true multi-omics integration remains scarce, leaving systems-level mechanisms of disease severity and host–pathogen interactions unresolved. High-throughput omics technologies have greatly advanced the study of hantavirus–host interactions. This review summarizes genomic, transcriptomic, proteomic, metabolomic, and AI-enabled approaches in hantavirus research. Genomic studies have clarified viral diversity, evolution, and reassortment, while transcriptomics has identified regulatory networks governing endothelial and innate immune responses. Proteomics has revealed host proteins involved in immune regulation, endothelial dysfunction, and potential therapeutic targeting, whereas metabolomics indicates substantial metabolic reprogramming during infection, although dedicated studies remain limited. AI-based approaches are increasingly applied to outbreak prediction, surveillance, and risk modeling. Drawing on successful multi-omics frameworks developed for other viral infections, we discuss opportunities to improve biomarker discovery, surveillance, therapeutic development, and future precision medicine strategies for hantavirus infections.

## 1. Introduction

Hantaviruses are an important and emerging global public health threat. Hantaviruses were first recognized in the 1970s after earlier outbreaks of Korean hemorrhagic fever, notably among troops during the Korean War, and have since proven to be remarkably capable of expanding geographically, adapting ecologically, and diversifying genetically [1,2]. These viruses cause two major clinical syndromes, Hemorrhagic Fever with Renal Syndrome (HFRS) in Eurasia and Hantavirus Cardiopulmonary Syndrome (HCPS) [3,4,5,6] in the Americas, with case fatality rates of up to 40%; however, Seoul virus is globally distributed, and clinical presentations can overlap [7,8]. The genus *Orthohantavirus* comprises 38 species representing 60 distinct viruses, although only a subset is associated with human disease [9]. The medically important hantaviruses discussed or mentioned in specific examples throughout this review include Hantaan virus (HTNV), Seoul virus (SEOV), Puumala virus (PUUV), Sin Nombre virus (SNV), Andes virus (ANDV), Juquitiba virus (JUQV), and Tula virus (TULV), all of which belong to the genus *Orthohantavirus*. These viruses differ in their geographic distribution, reservoir hosts, and pathogenic potential, but collectively represent the principal hantaviruses examined in omics studies. Hantaviruses have been identified worldwide, with more than 100,000 cases reported annually, most of which occur in China, Russia, Scandinavia, and the Balkans [2]. In the Americas, HCPS is less common but more deadly, with most reported cases in the United States, Chile, Argentina, and Brazil [10]. Sporadic but significant cluster outbreaks in Europe and South America in recent years have highlighted that hantavirus infection remains an active and unpredictable public health threat rather than a historically resolved concern [11]. Hantavirus remains endemic in different parts of the world. While cases tend to occur sporadically, clusters of infections are occasionally observed, complicating patient care and public health responses. Transmission of hantavirus to humans is primarily from animals, mainly through direct or aerosol exposure to infected rodent excreta. For human-pathogenic orthohantaviruses, the principal reservoir hosts are rodents, particularly muroid rodents, including cricetid subfamilies such as *Arvicolinae*, *Neotominae*, and *Sigmodontinae* [12]. More broadly, the genus *Orthohantavirus* also includes viruses associated with soricid and talpid eulipotyphla, such as shrews and moles, whereas the wider family *Hantaviridae* includes still greater host diversity [13]. However, established human disease is primarily associated with rodent-borne orthohantaviruses. Each hantavirus species often maintains a relatively specific relationship with its primary reservoir host, a pattern consistent with long-term host–virus coevolution and often referred to as codivergence [14]. Infected reservoir hosts generally remain asymptomatic, sustaining persistent infection and viral shedding into the environment [15]. This asymptomatic carrier state, combined with the wide geographic range of reservoir populations and their proximity to human habitats, creates an inherently dynamic spillover risk that is difficult to predict or control [16]. Clinical manifestations span an extraordinarily wide spectrum, from entirely asymptomatic seroconversion to rapidly fatal multiorgan failure. HFRS progresses through febrile, hypotensive, oliguric, polyuric stages and then recovery. The severe form is complicated by acute kidney injury, hemorrhage, and shock [17]. In contrast, HCPS has a sudden cardiopulmonary phase with noncardiogenic pulmonary edema and hemodynamic compromise, which accounts for its disproportionately high mortality [18]. The determinants of this clinical heterogeneity, such as host genetic polymorphisms in immune-regulatory genes, viral genotype, infectious dose, and immunopathological mechanisms such as T-cell-mediated endothelial injury, are still incompletely understood and constitute a central unresolved question in hantavirus pathobiology [19].

While established approaches have paved the way for hantavirus research, they lack the ability to depict the full complexity of viral pathogenesis, host–pathogen interactions, and disease heterogeneity. In virological and molecular epidemiology studies, individual molecular markers or pathways are usually studied separately. This has resulted in a compartmentalized understanding of the systemic biological processes underlying hantavirus infection and disease severity. This reductionist perspective has restricted progress in the development of broad-spectrum antivirals, the identification of reliable prognostic biomarkers, and the understanding of the mechanistic basis for variable clinical outcomes across species and host populations. Furthermore, the lack of comprehensive molecular profiles has limited the rational design of treatment interventions along with precision medicine approaches in hantavirus management.

Importantly, there is no widely accepted antiviral therapy or prophylactic vaccine currently available globally for hantavirus infection [20]. Among the antiviral agents investigated, ribavirin and favipiravir target viral RNA synthesis through distinct mechanisms: ribavirin depletes intracellular GTP pools, whereas favipiravir acts as an RNA-dependent RNA polymerase (RdRp) chain-terminating analogue and has recently been explored experimentally for the treatment of hantavirus infection [21]. However, ribavirin has shown limited or uncertain efficacy in early HFRS and no proven benefit in HCPS, with considerable toxicity [21,22].

Inactivated hantavirus vaccines are licensed or used regionally in China and South Korea, but no broadly approved antiviral therapy exists for use in the US, Europe, or most other regions, and the cross-protective breadth of these vaccines against diverse viral strains remains uncertain [20,23]. Supportive care, such as fluid management, renal replacement therapy, and extracorporeal membrane oxygenation in severe HCPS, therefore, remains the cornerstone of clinical management [24,25]. This profound therapeutic gap reveals the urgent need for mechanistically informed drug targets and validated prognostic biomarkers, both of which multi-omics approaches are well positioned to identify.

Beyond these core themes, progress across the broader biotechnological landscape continues to illuminate the technological and conceptual shifts driving contemporary therapeutic innovation. Notably, advances include the development of supramolecular metallogel systems engineered for medical applications, illustrating how emerging materials science can intersect with AI-guided antiviral discovery [26], as well as continued research on naturally derived molecules investigated in other disease settings [27,28]. Further progress has been made in elucidating the mechanisms underlying intracellular parasitic and viral infections and in evaluating their broader biological impact [29,30], along with advances in vaccination [31], precision therapies based on bioactive molecules, multi-omics methodologies [32], and modern drug-repurposing strategies [33]. Moreover, recent studies have deepened insight into protein-folding mechanisms in disease [34] and supported the development of novel anti-infective molecular scaffolds [35,36,37]. These developments are further reinforced by advances in biomacromolecular dynamics [38,39,40,41,42,43,44,45,46,47], computationally supported biological analysis [48,49], and the development of nature-inspired agents targeting disease-associated proteins [50,51]. These advances also extend to the valorization of natural bioresources for the development of functional, value-added products [52,53,54,55,56,57,58,59,60,61,62], as well as progress in state-of-the-art analytical chemistry methodologies [63,64]. The development of multi-omics technologies, including genomics, transcriptomics, proteomics, and metabolomics, among others, has transformed our capacity to simultaneously probe biological systems at multiple levels of molecular organization [65]. Herein, we assess how genomics, transcriptomics, proteomics, and metabolomics have collectively characterized viral genetic evolution, host transcriptional responses, circulating protein signatures, and metabolic alterations occurring during hantavirus infection. This systems-level view provides valuable insight into the changing networks of molecular interactions that control viral replication, innate immune activation, tissue damage, and disease progression. The availability of these integrated datasets offers the potential to identify novel therapeutic targets, to discover host-derived biomarkers that predict clinical outcomes, and to illuminate mechanistic pathways that drive pathogenesis across diverse viral species and epidemiological settings.

The use of artificial intelligence (AI) and machine learning (ML) algorithms with multi-omics data represents a paradigm shift in our ability to extract actionable biological insights from large-scale datasets [66]. High-level computational models may discover complex patterns, nonlinear associations, and predictive signatures that would otherwise remain obscured by conventional statistical analysis. These AI-based approaches support the development of risk stratification models, the prediction of therapeutic response, and the discovery of previously unknown associations among molecular features and clinical phenotypes [67,68,69]. Moreover, AI-augmented surveillance systems can improve the early detection of hantavirus circulation in animal reservoirs and deliver real-time epidemiological intelligence to support public health decision-making.

Hence, this review integrates recent advances in applying integrative multi-omics technologies to hantavirus research, with particular emphasis on understanding viral evolution, characterizing host immune and metabolic responses, and deploying AI-driven frameworks for surveillance and therapeutic discovery. In more detail, we discuss the technical foundations and analytical strategies underlying each omics modality, examine landmark studies demonstrating the utility of multi-omics integration, address current methodological challenges, and outline emerging perspectives for future research directions. By consolidating knowledge across these quickly evolving domains, this work intends to offer a comprehensive aid for researchers, clinicians, and public health professionals attempting to advance hantavirus science through systems-level approaches that ultimately advance patient outcomes and strengthen outbreak preparedness.

### Methods

A structured literature-based approach was used to prepare this review on integrative multi-omics in hantavirus research. Relevant evidence was collected from PubMed, Scopus, Web of Science, Google Scholar, public omics resources, genomic databases, and selected surveillance materials. The search covered studies published between 1998 and 2026.

Search terms included “hantavirus,” “orthohantavirus,” “HFRS,” “HCPS,” “HPS,” “genomics,” “transcriptomics,” “proteomics,” “metabolomics,” “multi-omics,” “host response,” “viral evolution,” “reassortment,” “machine learning,” “artificial intelligence,” and “biomarker discovery.” Species-specific terms such as “Hantaan virus,” “Puumala virus,” “Seoul virus,” “Andes virus,” and “Sin Nombre virus” were also used.

Eligible sources included original research articles, reviews, experimental studies, computational analyses, omics-based investigations, and relevant surveillance reports addressing hantavirus biology, evolution, host response, or translational applications. Non-scientific materials, unrelated viral studies, opinion-based papers without supporting data, and withdrawn or retracted publications were excluded. The included literature was then organized around genomic evolution, reassortment and recombination, host transcriptional responses, proteomic and metabolomic changes, and future AI-driven multi-omics applications.

## 2. Genomics and Evolutionary Dynamics of Hantaviruses

Hantavirus evolution is shaped by the interaction between a segmented RNA genome, strong functional constraints, and ecological opportunities for viral diversification. Understanding genome organization, molecular-clock dynamics, reassortment, recombination, host-range determinants, and translational consequences provides an important framework for interpreting hantavirus emergence, pathogenicity, and countermeasure development.

### 2.1. Genome Organization and Structural Architecture

**Figure 1:**
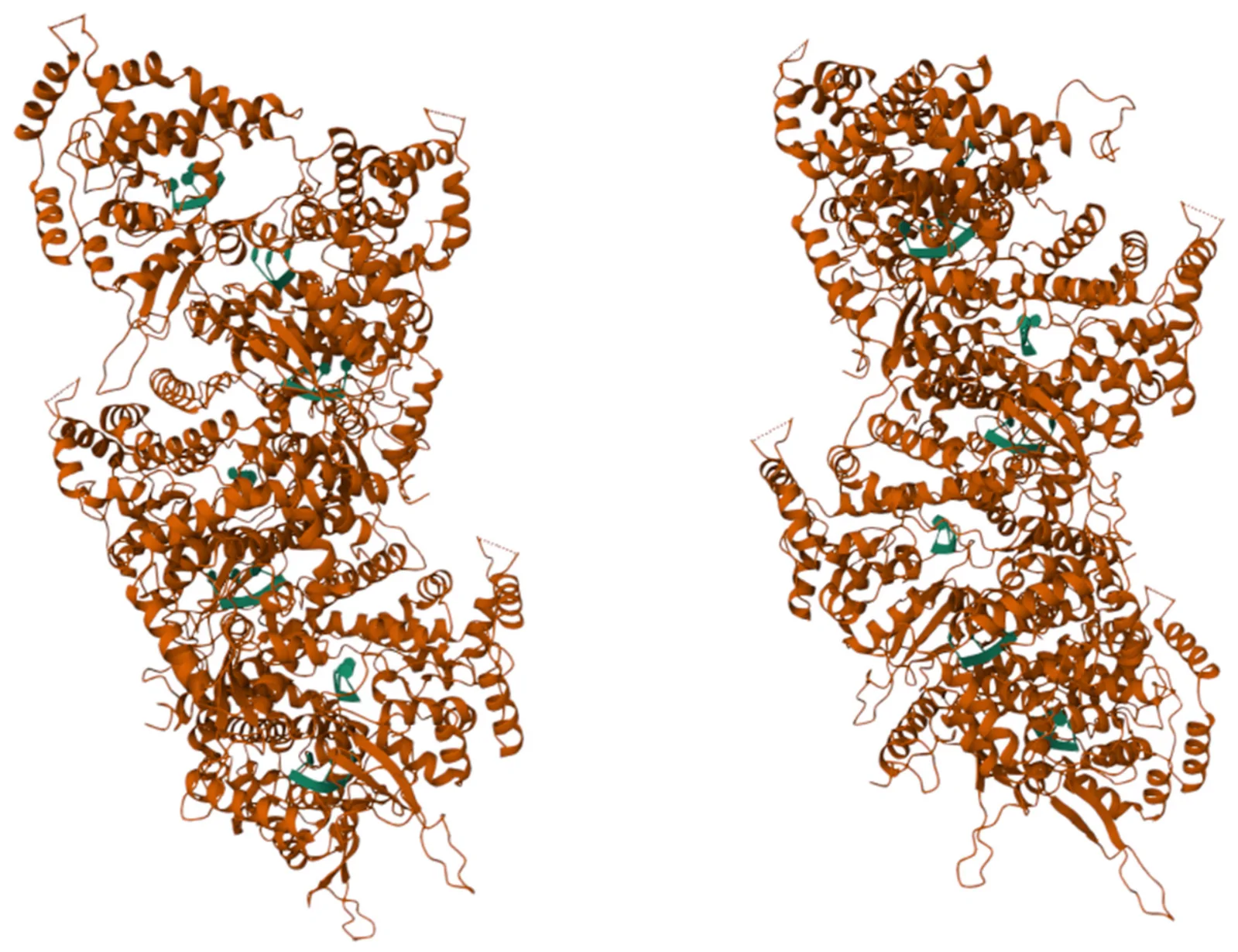
Three-dimensional views of the *Orthohantavirus hantanense* nucleocapsid bound to double-helical RNA elements (shown in green). The structure was obtained from the Protein Data Bank (PDB ID: 6I2N) [70] and visualized using the RCSB PDB 3D viewer (https://www.rcsb.org/3d-view/6I2N/1 accessed on 27 June 2026).

Hantaviruses have a tripartite single-stranded negative-sense RNA genome composed of three functionally distinct segments, small (S), medium (M), and large (L), with a total length of about 11.1–12.3 kb [9]. The S segment encodes the nucleocapsid (N) protein, the major virion structural protein (Figure 1).

The N protein encapsidates and protects the three genomic RNAs, generating three viral ribonucleoprotein complexes [71]. The M segment encodes a glycoprotein precursor that is processed into two envelope glycoproteins, Gn and Gc, that mediate host cell attachment and membrane fusion [72]. The L segment encodes the viral RNA-dependent RNA polymerase (RdRp) that orchestrates genome replication and transcription [73]. The S segment may also contain an additional overlapping open reading frame in some hantavirus species, which may encode a non-structural (NS) protein. However, NS protein expression is not universal among lineages and seems to be more important for innate immune antagonism than for core replication [74]. Complementary terminal sequences at the ends of the segments are conserved and form promoter elements recognized by the L protein. The 5′ RNA adopts a hook conformation, and the distal termini form a duplex promoter architecture. Structural studies of Hantaan polymerase have revealed promoter-induced activation, prime-and-realign initiation, and conformational changes that are critical for replication [75]. Minigenome studies also indicate that the S-segment 3′ untranslated region (UTR) architecture modulates replication efficiency. Although the tripartite genome organization is conserved among hantaviruses, segment lengths and coding capacity vary among species [76,77]. A comparative summary of representative hantaviruses is provided in Table 1.

### 2.2. Evolutionary Rates and Selection Dynamics

Recent short-term hantavirus molecular-clock estimates fall within an operationally useful range of approximately 1.6 × 10^−4^ to 1.16 × 10^−3^ substitutions per site per year, spanning longitudinal HTNV estimates and TULV S-segment fixed-local-clock estimates [94,95]. Yet this apparent range masks deeper complexities that shape epidemiological interpretation. The segment-specific architecture of the hantavirus genome causes predictable rate heterogeneity. Polymerase-encoding L segments evolve most conservatively (1.6–5.1 × 10^−4^), followed by nucleocapsid protein (2.6–3.1 × 10^−4^), while surface glycoproteins, especially Gn, show faster turnover (3.3–6.6 × 10^−4^), with Gc displaying an intermediate rate (2.4–3.8 × 10^−4^). This gradient reflects the functional architecture of each protein, with polymerase constraints (precision in RNA synthesis, template recognition) imposing stronger evolutionary brakes than glycoprotein domains involved in receptor engagement and immune evasion [94]. Critically, inferred rates are exquisitely sensitive to sampling design, temporal coverage, and clade composition [96]. Consequently, outbreak investigations relying on standard clock estimates without local calibration may risk systematic dating errors that cascade through transmission reconstruction. This implies that molecular-clock inference in hantaviruses requires explicit hierarchical modeling that partitions rate heterogeneity by geography, host population history, and viral lineage rather than applying single global estimates [97]. Despite detectable sequence flux, hantavirus evolution operates under stringent negative selection. dN/dS ratios, which represent the ratio of nonsynonymous to synonymous substitution rates, remain below 0.1 across major structural and regulatory proteins and indicate strong purifying selection, suggesting that nonsynonymous substitutions are strongly constrained. The predominance of this constraint across the L, Gn, Gc, and N proteins suggests a highly constrained fitness landscape, in which only a limited range of amino acid variation is tolerated [94].

The distinction between synonymous and nonsynonymous diversity becomes particularly informative when nucleotide-level sequence variation decouples from functional change, a pattern documented in contemporary rodent surveys using nanopore sequencing [94]. Populations harboring high nucleotide diversity frequently display minimal amino-acid polymorphism, revealing that synonymous sites serve as molecular slack that accommodates genetic drift without perturbing viral function. Positive selection, when detected, is rare and localized [98]. Evidence for adaptively driven change in Gn exists but remains subtle and often tissue- or host-specific rather than a pandemic-scale phenomenon. This observation suggests that hantavirus emergence is not typically driven by rapid adaptive walks across the fitness landscape but rather by ecological juxtaposition (rodent range expansion, human encroachment) of pre-existing, quasi-fixed viral populations [16].

### 2.3. Reassortment and Recombination as Sources of Genetic Novelty

**Figure 2:**
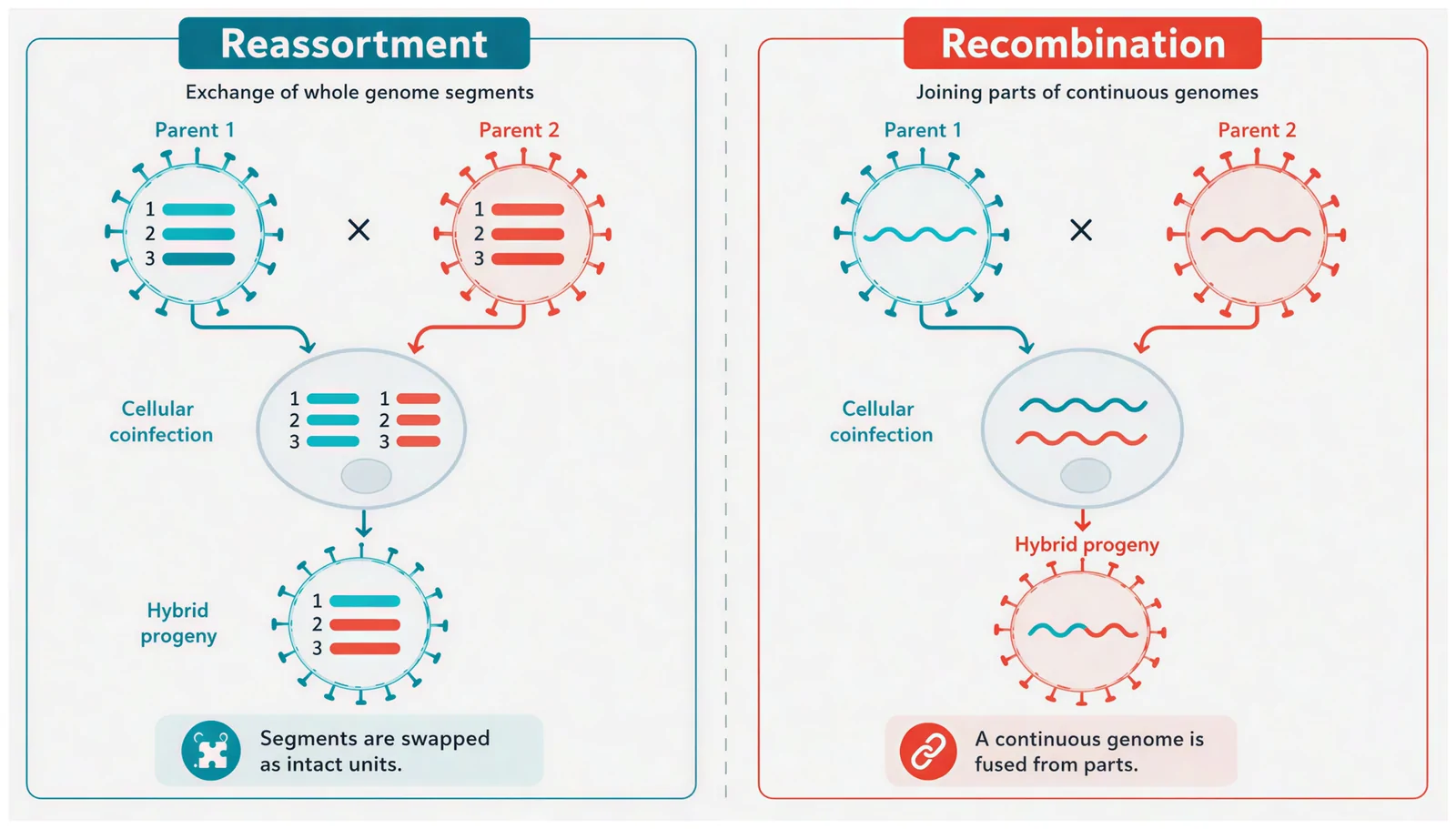
Schematic comparison of reassortment and recombination in hantaviruses. Reassortment involves the exchange of intact genomic segments during cellular coinfection, whereas recombination generates hybrid RNA molecules through the joining of sequences derived from different parental genomes. Created by the authors using Canva (https://www.canva.com accessed on 17 July 2026).

If point mutation is constrained, reassortment, which is defined as the exchange of intact genome segments during coinfection, emerges as an important mechanism for generating genetic and functional novelty at epidemiologically relevant timescales [99]. The mechanistic prerequisite is that two or more hantavirus variants infect the same host and, critically, the same cell, where L, M, and S ribonucleoprotein segments can be independently packaged into progeny virions [100]. Ecologically, this requirement is far from trivial, and it demands geographic overlap, high host population density, and repeated local introductions that create coinfection windows [101]. Figure 2 illustrates the mechanistic distinction between whole-segment reassortment and within-segment recombination during cellular coinfection.

Yet reassortment is demonstrably common. Regional surveillance across Russia, Korea, and contemporaneous studies document reassortant L-M, L-S, and M-S constellations in high-incidence zones, with genetically distant segments combined in single isolates while retaining compatibility and transmissibility [94,101,102]. The 2025 high-fidelity nanopore surveys in Korea identified additional reassortant candidates circulating in contemporary wild rodent populations, suggesting that reassortment is not a historical artifact but an ongoing evolutionary process. So, the diversity observed in field populations is not purely vertical but also combinatorial, a distinction with profound consequences for tracing outbreak origins and predicting viral phenotypes [103].

Critically, reassortment is ecologically contingent. Its frequency and impact are highest in geographic regions where multiple viral lineages maintain overlapping ranges and reservoir hosts exist in sufficient density to support repeated coinfections [104]. Sparse, isolated rodent populations may harbor diverse viruses but are expected to generate reassortants less frequently because opportunities for coinfection are limited. This heterogeneity in reassortment opportunity structure means that reassortment-driven emergence is a regional, not universal, phenomenon. It amplifies genetic novelty where conditions permit, but remains silent in fragmented ecosystems [101].

Hantaviruses historically provided molecular evidence for template-switching recombination within RNA segments, particularly in HTNV and Tula virus, marking them as instructive examples among negative-sense RNA viruses where such events are mechanistically plausible and occasionally documented [105,106,107]. Yet recent literature reveals an asymmetry in that reassortment dominates contemporary genomic surveys, while recombination-driven diversity remains inconsistently detected and modest in magnitude. Many recent high-coverage studies do not recover recombination as a major driver of population-level genetic structure relative to segment exchange [98,101].

Recombination is mechanistically credible, historically documented, and biochemically plausible given the hantavirus replication strategy. However, its population-level importance remains uncertain [98,101]. Reassortment, by contrast, is established, recurrent, and epidemiologically consequential [103]. This distinction is not semantic; it alters surveillance strategy. If reassortment dominates, monitoring phylogenetic incongruence across segments becomes a priority. If recombination is rarer, segment-by-segment phylogenetic tracking may suffice [101,108].

### 2.4. Host Range and Virulence Determinants

The M segment of the viral RNA genome encodes the two surface glycoproteins Gn and Gc, which mediate receptor engagement and membrane fusion and are major determinants of viral entry and host range [72]. However, disease phenotype is multifactorial and also depends on S-segment immune antagonism, L-segment replication functions, and host immune and genetic context [76]. This central role is not uniform across the hantavirus phylogeny and is most precisely defined in New World pathogens. For Andes and Sin Nombre viruses, host entry depends on protocadherin-1 (PCDH1), a cell adhesion molecule. Structural mapping has revealed that the Gn/Gc-PCDH1 interface is tightly constrained; even single-residue changes in the host receptor can measurably alter viral specificity and disease outcome in experimental models [109]. By contrast, β3 integrin, β1 integrin, and decay-accelerating factor (DAF), which have also been proposed as hantavirus entry factors, are neither universally nor consistently required across New World hantavirus entry pathways [110]. Functionally, this implies that New World hantavirus tropism appears to be strongly influenced by Gn/Gc determinants and their cognate host receptor, creating a system where genetic variation in either viral or host sequence can shift host specificity and clinical severity [109]. Old World viruses (HTNV, PUUV, TULV) likely operate under different constraints; their dominant entry determinants remain incompletely mapped, suggesting either functional redundancy, reliance on alternative receptors, or mechanistic differences relative to New World taxa [110]. This gap constrains predictive models of zoonotic spillover and limits rational vaccine and therapeutic design for Eurasian hantavirus pathogens.

Identifying the genetic basis of hantavirus pathogenesis has advanced significantly but remains incomplete. Comparative genomics of Andes virus strains differing in severity identified four amino-acid substitutions associated with cell-culture adaptation and attenuated disease in hamster models; the same analysis implicated additional candidate changes potentially relevant to person-to-person transmission, though reverse-genetic verification remains outstanding [111,112]. In the *Juquitiba virus* (South American), a single Gn mutation (V504I) demonstrably reduced pseudotype entry efficiency, providing a functional link between sequence change and entry phenotype [113]. The existence of such examples proves that causality is achievable but also highlights its rarity; most genomic candidates for virulence-associated determinants lack experimental validation. The accessory, non-structural S-segment layer provides a second axis of virulence control. The NS protein, when expressed, acts as a potent interferon antagonist [114]. Andes NSs inhibit MAVS signaling, a critical hub in intracellular antiviral sensing, while PUUV NSs and GPC together suppress RIG-I-dependent type-I interferon responses [115]. This makes the S segment an underappreciated determinant of host permissiveness and clinical severity. Mutations that enhance interferon evasion could potentially increase virulence, yet population-level evidence linking S-segment variants to disease outcomes is sparse. Screening for causal NS variants represents a clear priority for the field.

Polymerase-driven virulence determinants are even less mature experimentally, despite mechanistic plausibility. The L protein performs multiple precision functions, including promoter recognition and binding, endonuclease activity involved in cap-snatching, and oligomerization into functional replication complexes; during transcription, L mediates initiation through cap-snatching by using capped host-derived RNA fragments as primers [71,116]. Each function is a potential antiviral target and a potential virulence determinant; mutations that destabilize polymerase function could attenuate disease, while mutations enhancing processivity or evasion of cellular surveillance may amplify pathogenesis. However, systems-level analysis linking L-protein sequence variation to clinical or molecular phenotypes has not yet been performed at the population scale.

### 2.5. Translational Implications for Diagnostics, Vaccines, and Antivirals

Genomic diversity, evolutionary constraint, and functional mapping directly inform countermeasure design. The segment-to-segment variation documented above argues against reliance on diagnostics built on narrow historical reference panels or single loci. Instead, the field has adopted multiplex reverse-transcription quantitative PCR (RT-qPCR), designed with lineage-aware primer sets that track regional diversity and require sequence confirmation for definitive identification [117]. Hybrid-capture panels spanning the orthohantavirus genus, high-fidelity long-read nanopore sequencing for rapid outbreak tracing, and amplification-free CRISPR-Cas13 detection now complement conventional PCR, collectively reducing both false negatives and false-positive misidentification that plagued earlier single-primer approaches [118,119,120]. On the other hand, vaccine development exemplifies translational integration, and rational design has shifted from older inactivated whole-virus products toward structure-guided Gn/Gc immunogens, DNA-based platforms encoding prefusion-stabilized glycoproteins, and mRNA-lipid nanoparticle (mRNA-LNP) boosters [121,122]. Prefusion-stabilized HTNV glycoprotein DNA and mRNA vaccines elicit potent neutralizing antibody responses and confer protective immunity in animal models, directly utilizing the structural knowledge of Gn/Gc receptor engagement [121].

Remarkably, antiviral discovery has converged on the L protein as an important molecular target. Establishment of BSL-2 minigenome systems, which use reporter genome analogs to model hantavirus transcription and replication without producing infectious virus, now enables systematic screening of polymerase-directed compounds, including ribavirin, remdesivir, baloxavir, and the nucleoside analog 2′-deoxy-2′-fluorocytidine. Each inhibits distinct polymerase functions (nucleotide incorporation, capping, cap-snatching), providing mechanistic diversity and reducing the likelihood of resistance emergence [77]. The convergence of genomic understanding of polymerase structure and function with minigenome-based antiviral screening represents a promising translational pathway from basic research to clinical candidate identification; however, these findings remain preclinical and require validation in infectious-virus systems, animal models, and clinical studies.

## 3. Host Response Profiling Through Transcriptomics

Transcriptomic approaches have provided important insights into the molecular mechanisms of hantavirus pathogenesis by revealing dynamic changes in host gene expression during the course of infection. Bulk, single-cell, and non-coding RNA analyses have revealed key regulatory networks in antiviral responses, immune dysregulation, endothelial dysfunction, and disease progression. The following sections review major transcriptomic findings across different cellular, temporal, and host-specific contexts.

### 3.1. Multi-Layer Transcriptional Networks in Hantavirus-Infected Endothelium

Hantavirus infection induces synchronized multi-layer transcriptional networks rather than isolated differential expression patterns. Profiling of HTNV-infected endothelial cells identified 70 differentially expressed circular RNAs, 66 miRNAs, and 788 mRNAs. Functional validation showed that type I interferon signaling, antiviral defense pathways, and innate immune responses are the major transcriptional signatures. Importantly, these results demonstrated that regulatory control is not single-gene-based but rather a function of connected RNA networks. A key example is the competing endogenous RNA axis circ_0000479/miR-149-5p/RIG-I, where knockdown of circ_0000479 enhances viral nucleoprotein expression, implying circular RNAs act as transcriptional regulators of viral replication. Further validation identified a number of differentially expressed RNAs, including miR-149-5p, miR-330-5p, miR-411-3p, CMPK2, PARP10, and GBP1, that promote or inhibit HTNV replication. These results established that the antiviral transcriptome functions through multilayered regulatory checkpoints encompassing non-coding and protein-coding genes [123].

### 3.2. Single-Cell Transcriptomics and Discovery of Unexpected Cellular States

Single-cell transcriptomics has moved beyond population-averaged signatures to resolve disease-relevant cellular states and identify unexpected cell populations [124]. Profiling of peripheral blood mononuclear cells from HFRS patients revealed that circulating RBC clusters previously assumed to be contaminating mature erythrocytes are in fact nucleated red blood cells (NRBCs), a previously undercharacterized compartment that expresses a remarkable dual phenotype. These NRBCs express cytotoxic effector-associated genes (CD3E, granulysin, granzymes A/B/H/K) alongside type I interferon response genes (IRF1, IFITM2, IFITM3, FAM46C, LY6E) and MHC class I and II molecules. Notably, NRBC abundance correlates directly with disease severity and viral load, suggesting these cells represent a previously overlooked link between erythroid dysregulation and immune activation. Pseudotime analysis revealed a developmental trajectory from early-stage progenitor-like cells to late-stage erythroblasts, and clinical analysis linked this NRBC phenotype to impairment in folate metabolism and reduced vitamin B12/B9 levels, indicating that HFRS-associated erythroid abnormalities have distinct metabolic underpinnings beyond viral cytotoxicity alone. Functional validation demonstrated that approximately 10% of CD71^+^CD235a^+^ NRBCs and 6% of mature CD71^−^CD235a^+^ RBCs express αvβ3, a proposed hantavirus entry factor, and in vitro HTNV infection of blood cells resulted in nucleoprotein detection in >10% of RBCs at 72 h post-infection, establishing that infected erythroid cells may represent a previously underrecognized infected blood-cell compartment [125].

Reanalysis of the same single-cell dataset resolved 33 clusters encompassing 11 distinct immune cell types, revealing reduced T-cell proportions, altered monocyte states, and extensive cell–cell communication networks mediated by MIF, CD70, and GALECTIN families. Weighted gene coexpression network analysis and trajectory analysis identified T- and B-cell coexpression modules linked to HFRS pathogenesis, with elevated GZMH and GZMK expression in effector CD8^+^ T cells, naive CD4^+^ T cells, and naive B cells. This transcriptomic single-cell atlas (GSE161354) is now a reusable resource for the detailed characterization of hantavirus-induced immune dysfunction, and highlights the power of single-cell approaches to reveal heterogeneity masked by bulk analyses [126].

### 3.3. Cell-Type-Specific Responses: Neutrophil and Granulocyte Dynamics

Analysis of separated polymorphonuclear neutrophils (PMNs) and low-density granulocyte (LDG) subsets in Puumala virus-caused HFRS demonstrates how targeted cell-type transcriptomics can reveal biology invisible to both bulk and even single-cell approaches. While conventional PMNs exhibited activation of antiviral pathways, circulating LDGs were significantly increased in frequency following acute PUUV-HFRS infection. Cell surface marker analysis revealed that PUUV-associated LDGs are primarily immature neutrophils, indicating increased bone marrow production rather than altered differentiation. Notably, both LDG frequency and peripheral blood left shift are associated with thrombocytopenia severity, establishing neutrophil maturation state as one of the clearest transcriptome-linked disease severity correlates in orthohantavirus infection. In contrast to COVID-19-associated LDGs, PUUV LDGs showed little immunosuppressive activity, emphasizing that LDG phenotypes are not a generalized consequence of acute infection but are virus- and kinetics-dependent, an important distinction for understanding cell-type-specific responses across viral pathogens [127].

### 3.4. Phase-Resolved Transcriptomics and Disease Progression Dynamics

It has been shown that static case–control designs may not fully capture disease progression using phase-resolved transcriptomic analysis [128]. Longitudinal study of peripheral blood mononuclear cells in the oliguric, diuretic, and convalescent phases of HFRS identified 38 genes with consistent expression changes in all three recovery phases. Among upregulated genes, eight demonstrated monotonic increases, including immune checkpoint genes (CD83, NR4A1) alongside heat-shock proteins (DNAJB1, HSPA1B, HSPA1A) and other recovery-associated factors (FAM71A, FCRL6, DUSP8); conversely, four genes, including antiviral/immunomodulatory factors (IFI27, RNASE2) and plasma cell markers (MZB1, IGKV1-33), showed monotonic decreases. Gene Ontology analysis revealed a coherent temporal logic: transitioning from the oliguric to diuretic phase, enriched tissue morphogenesis and renal system development terms were associated with upregulated genes, and enriched antimicrobial humoral response and myeloid leukocyte-mediated immunity terms were associated with downregulated genes. During the transition from the diuretic to the convalescent phase, immune checkpoint activation was observed, along with increased expression of genes involved in translational initiation and protein targeting to the endoplasmic reticulum. In contrast, phagocytosis, immunoglobulin production, and complement activation were decreased. Deconvolution of immune cells showed that M2 macrophages and resting memory CD4+ T cells increased in a stepwise manner across phases, suggesting a coordinated transition from inflammatory antimicrobial responses to anti-inflammatory and immune-memory phenotypes [129]. These results show that phase-resolved sampling resolves disease dynamics and identifies gene signatures that stratify recovery trajectories, a principle that should inform the future design of transcriptomic studies in acute viral infections.

### 3.5. Cross-Species Transcriptomics: Human Pathogenesis vs. Reservoir Host Adaptation

Cross-species transcriptomics has revealed an important distinction in how human and reservoir hosts respond to hantavirus infection, highlighting that viral persistence is not a failure of viral sensing but rather a regulated transcriptional reprogramming of host–pathogen crosstalk. Human lung microvascular endothelial cells infected with Seoul virus mount a delayed but robust type I interferon-dependent antiviral program characterized by canonical interferon-stimulated genes (MX2, RSAD2, CMPK2, IFIT1, MX1, IFI44L, OAS2, IFIT3, OASL, IFITM1). This response reflects classical antiviral surveillance and mounting of competitive effector responses against viral replication. In contrast, rat lung microvascular endothelium responds earlier with more efficient infection, but over time, its transcriptome undergoes a regulated shift from an antiviral state toward an immunotolerant state. This shift is mediated by a PD-L1, B2M (beta-2 microglobulin), and JAK2-focused interaction network that is predicted to suppress cytotoxic CD8^+^ T cell activation. Specifically, Cd274 (encoding PD-L1) is significantly upregulated 2.69-fold over mock at 12 days post-infection, B2m 1.93-fold, while Jak2 expression decreases 1.32-fold. These data support the hypothesis that this transcriptional rewiring represents an important strategy enabling viral persistence in which, after initial detection of infection through conserved pattern recognition, the endothelium actively recruits immune checkpoint pathways to prevent clearance [130].

**Figure 3:**
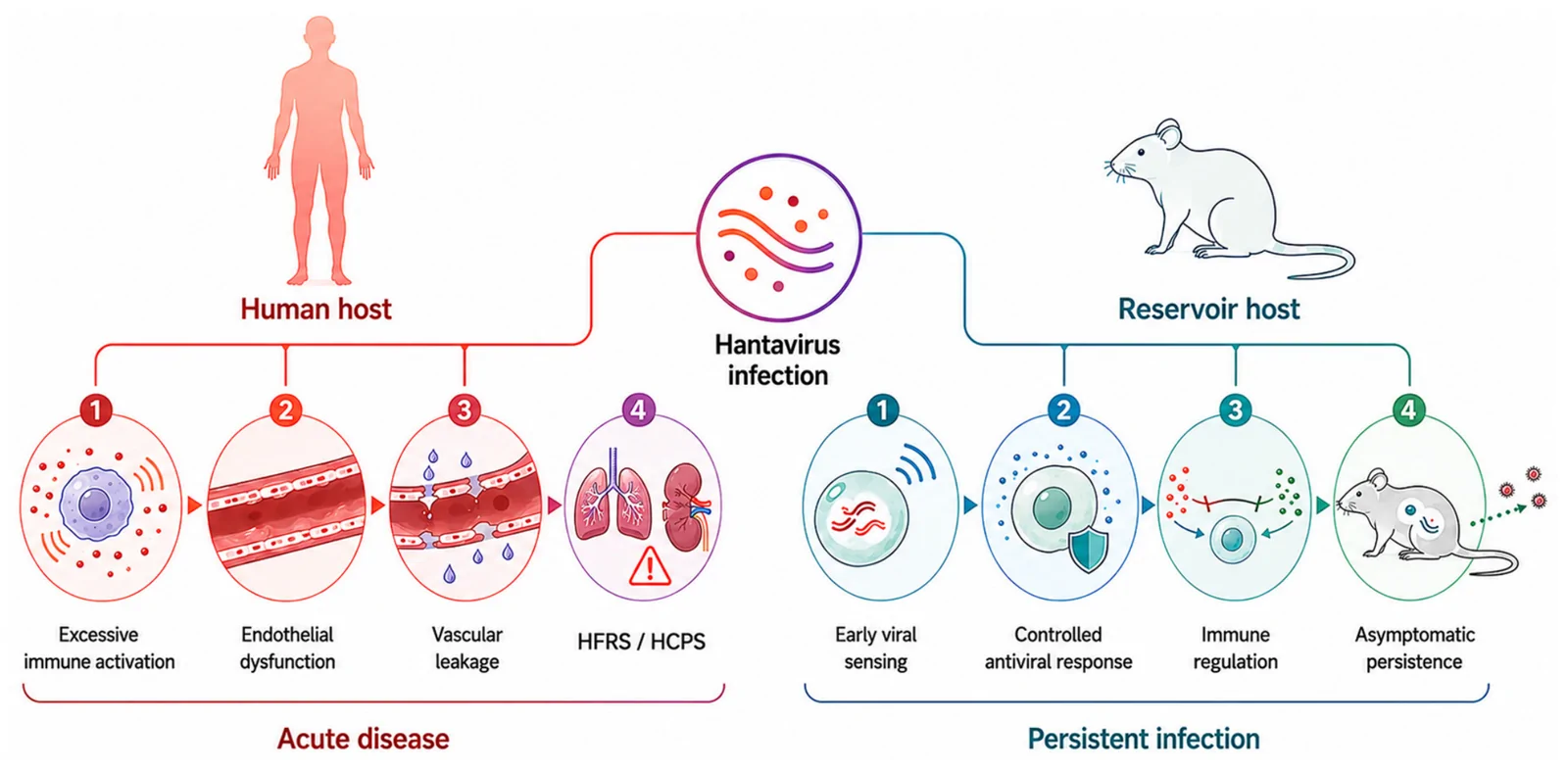
Divergent host responses to hantavirus infection. In humans, excessive immune activation promotes endothelial dysfunction, vascular leakage, and acute HFRS or HCPS, whereas regulated antiviral responses in reservoir hosts support asymptomatic viral persistence. Created by the authors using Canva (https://www.canva.com/, accessed on 17 July 2026).

A parallel principle emerges in macrophage biology. Examination of Hantaan virus-infected mouse and human macrophages reveals that Notch and NF-κB pathway crosstalk determines divergent infection outcomes. In both species, Notch receptors activated by HTNV enhance NF-κB signaling by recruiting IKKβ and p65, promoting inflammatory M1-like macrophage polarization. However, in mice but not humans, this Notch-mediated inflammation is timely restrained by murine-specific long noncoding RNAs transcribed by the Notch pathway in negative feedback loops. Notably, lnc-ip65 (lncRNA 30740.1) detaches p65 from the Notch receptor and inhibits p65 phosphorylation, rewiring macrophages from pro-inflammatory to pro-resolution phenotypes. Genetic ablation of lnc-ip65 results in destructive HTNV infection in mice, demonstrating that a single murine-specific noncoding RNA is critical for immune restraint and survival. The study linked this transcriptional switch to measurable immunometabolic changes, including alterations in mitochondrial function and metabolic reprogramming from M1 to M2 states [131]. These findings suggest a novel mechanistic framework in which viral persistence in reservoir hosts is not due to defective viral sensing or inferior adaptive immunity, but rather reflects species-specific transcriptional circuits, particularly noncoding RNAs that actively restrain inflammation after initial antiviral detection. This principle has important implications for understanding why humans progress to acute disease while reservoir rodents maintain asymptomatic persistence. One of the most mechanistically informative examinations of reservoir-host adaptation remains analysis of naturally infected long-tailed pygmy rice rats (the principal Andes virus reservoir), which distinguished one infected animal with approximately 50-fold higher viral burden (consistent with acute infection) from two animals with lower burdens (consistent with persistence). In persistently infected animals, differential expression revealed suppression within the JAK-STAT pathway (Stat5b, Ccnot1), elevation of caspase-1 (Casp1), elevation of RIG-I pathway-associated factors (Ppp1cc, Mff), and increased Fc-receptor-like transcripts. Given that caspase-1 and Stat5b activation pathways stimulate T follicular helper cell (TFH) development, these findings suggest that persistent infection is accompanied by adaptive immune tolerance through TFH differentiation, which is consistent with serological reports of TFH stimulation in deer mice experimentally infected with hantaviruses. By contrast, in the acutely infected rice rat, apoptotic pathway markers (Cox6a1) were elevated, and putative antiviral factors (Abcb1a, Fam46c, Spp1, Rxra, Rxrb, Trmp2, Trim58) were modulated while preproenkephalin (Prenk) transcripts were reduced, potentially reflective of altered T cell activation thresholds. This in vivo comparison between acute and persistent infection in naturally infected reservoir hosts provided direct evidence that transcriptional suppression of JAK-STAT signaling may be associated with hantavirus persistence, supporting observations from cell culture and cross-species comparisons [132]. These contrasting host-response trajectories, leading either to acute disease in humans or asymptomatic persistence in reservoir hosts, are conceptually summarized in Figure 3.

### 3.6. Virus- and Host-Species-Dependent Cellular Tropism Constraints

Paired human and bank vole cell transcriptomics from cells infected with three orthohantavirus species (PUUV, TULV, PHV) have revealed that viral tropism is determined by virus- and cell-type-dependent regulation of interferon responses and viral-cellular protein interactions. In bank vole cells, PUUV completed its replication cycle, TULV failed to establish a productive infection, and PHV replicated but did not produce infectious particles. Electron microscopy revealed divergent assembly failures: TULV particles accumulated at the plasma membrane, while PHV virions were sequestered in autophagic compartments. RNA-seq analysis combined with proteomics profiling revealed virus-specific regulation of interferon-stimulated genes and differential viral-cellular protein interactions, especially involving the viral nucleocapsid protein. These results indicate that tropism constraints are acting at the transcriptional, translational, and post-translational levels simultaneously, and suggest the need for multi-omics integration to fully resolve viral-host determinants of permissiveness [133].

### 3.7. Spatial Transcriptomics: An Emerging Frontier

Despite the wealth of bulk and single-cell transcriptomic studies, spatial transcriptomics remains an underdeveloped opportunity rather than an established investigative modality in hantavirus research. Yet, tissue-resolved spatial transcriptomic maps have not become routine practice in peer-reviewed hantavirus publications. Dedicated in vivo infection experiments followed by spatial transcriptomics could provide unprecedented insights into cellular heterogeneity and tissue-specific responses during hantavirus infection, particularly in organs subject to severe pathology (lungs, kidneys, endothelium). Such approaches would enable tissue-level resolution of cellular compartmentalization, immune infiltration dynamics, and site-specific transcriptional signatures that bulk and single-cell approaches fundamentally cannot distinguish [134,135]. The absence of spatial transcriptomic data thus represents a significant methodological gap in current hantavirus host-response profiling, addressing which would unlock tissue-resolved understanding of hantavirus pathogenesis.

## 4. Proteomics, Metabolomics, and Integrated Host–Virus Responses

**Figure 4:**
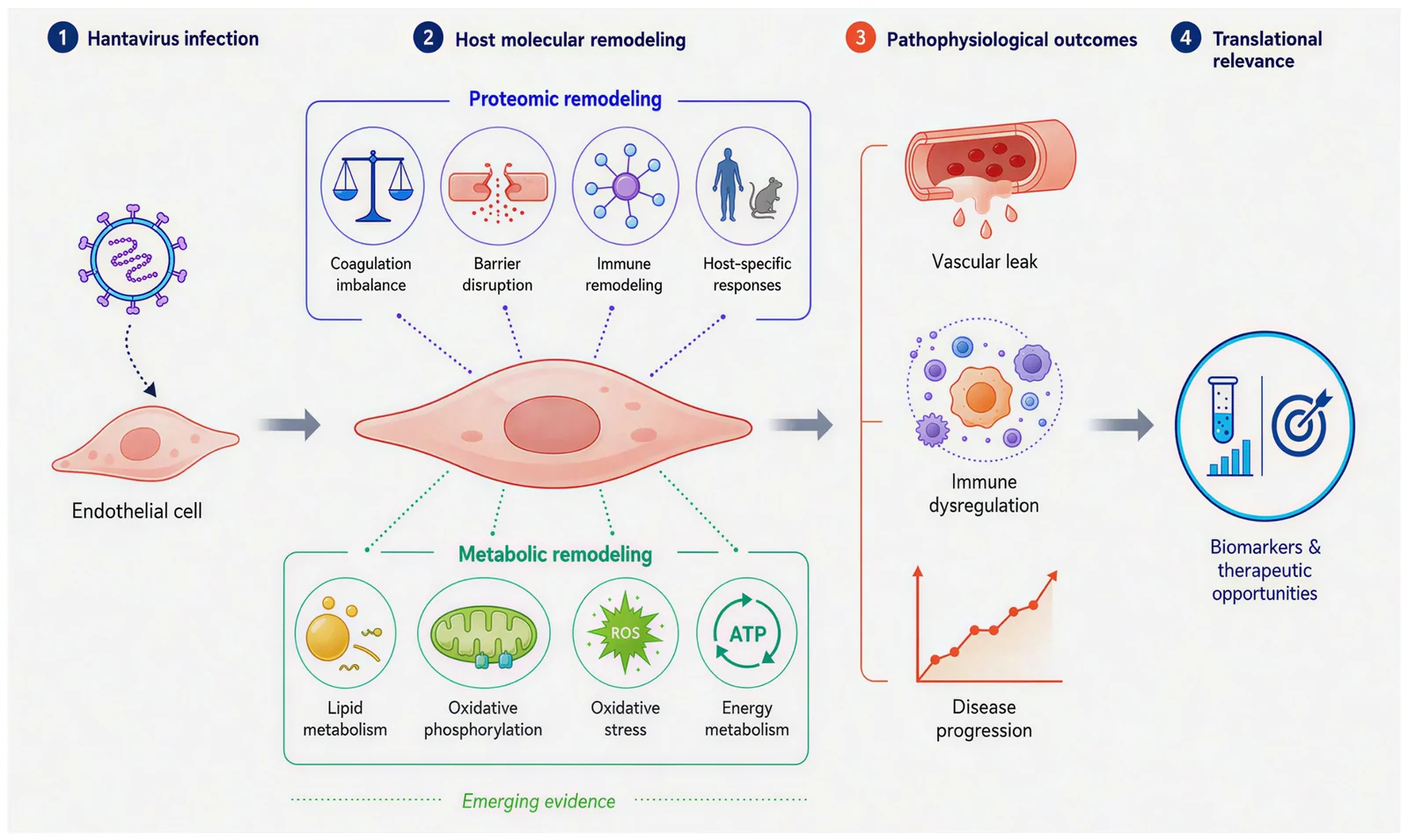
Proteomic and metabolic remodeling during hantavirus infection and its major pathophysiological and translational consequences. Hantavirus-induced changes in endothelial cells contribute to coagulation imbalance, barrier disruption, immune dysregulation, vascular leakage, and disease progression, while highlighting potential biomarkers and therapeutic targets. Created by the authors using Canva (https://www.canva.com/, accessed on 17 July 2026).

Through the identification of functional changes in host proteins and metabolic pathways during infection, proteomic and metabolomic approaches have broadened our understanding of hantavirus pathogenesis. These approaches have uncovered mechanisms associated with immune activation, vascular dysfunction, and metabolic reprogramming, providing important insights into disease progression and host–virus interactions. A schematic overview of the proteomic and metabolic remodeling induced by hantavirus infection and its major pathophysiological and translational consequences is presented in Figure 4.

### 4.1. Plasma Proteomics and the Coagulation-Fibrinolysis Axis in HCPS

Although proteomics studies of hantavirus pathogenesis remain limited compared to transcriptomic approaches, available investigations have provided critical mechanistic insights into the vascular dysfunction and coagulopathy central to severe disease [136]. Plasma proteomics of severe HCPS reveals a fibrinolytic-coagulation imbalance that tracks disease severity independently of viral burden. The simultaneous marked elevation of PAI-1, thrombin, and tPA in terminal cases is not simply a coagulation activation signature; it reflects a specific failure of antifibrinolytic regulation in which fibrinolytic potential is generated but functionally neutralized, since co-elevation of tPA and PAI-1 at high concentrations distinguishes severe from mild disease and is absent in survivors. This proteomic profile positions the coagulation-fibrinolysis axis as a mechanistic determinant of capillary integrity failure rather than an epiphenomenon of systemic inflammation [137]. The mechanistic picture extends further: PAI-1-refractory Urokinase plasminogen activator (uPA) activity and the concurrent absence of tPA activity characterize HCPS plasma, with P2Y_2_R upregulation and active uPA emerging as functional effectors of the procoagulant endothelial phenotype [138]. Upstream of these coagulation events, IL-6 trans-signaling drives VE-cadherin internalization via VEGFR2-dependent Src family kinase activation, elevating endothelial monolayer permeability and providing a cytokine-level mechanism that links immune activation directly to the adherens junction disruption that plasma proteomics had previously characterized at the protein level [139,140]. Together, these findings reframe the proximate cause of microvascular failure in HCPS, as endothelial adhesion molecule degradation and fibrinolytic dysregulation may contribute to vascular leak, rather than representing downstream consequences of uncontrolled viremia [137].

### 4.2. Cell-Based Proteomics and the Molecular Basis of Host Specificity

Comparative proteomic profiling across human and rodent cell lines infected with pathogenic, low-pathogenicity, and non-pathogenic orthohantaviruses demonstrates that infection outcome is determined at multiple discrete steps, including viral entry, replication efficiency, and particle assembly, and that these restrictions are both virus-strain-specific and cell-type-dependent [133]. In rodent reservoir cells, the intracellular environment is permissive for complete viral cycles, with suppressed proinflammatory and antiviral responses at sites of high viral replication [133,141]. Human endothelial cells, by contrast, mount robust innate immune activation, including type I interferon responses, RIG-I-like receptor pathway engagement, ISG overexpression, and antigen-presentation machinery upregulation that restricts viral propagation but simultaneously drives the vascular dysfunction and immunopathology characteristic of severe disease [19,141,142,143]. This species-specific dichotomy between productive tolerance and immunopathogenic restriction is not resolvable by transcriptomics alone, because it depends on host-viral protein interactions, post-translational states, and viral protein distribution that only proteomics and electron microscopy-guided virion tracking can directly interrogate [19,141]. A comprehensive mass spectrometry dataset capturing these host-restriction determinants across species boundaries is now publicly accessible via the ProteomeXchange/PRIDE repository (PXD032365), providing a foundational resource for the mechanistic dissection of pathogenic versus persistent infection [133,144].

### 4.3. Convergent Proteomics Signatures Across Sample Types and Platforms

Integrating across plasma and cell-based studies, four proteomics-grounded signatures of hantavirus pathogenesis emerge with consistency. First, fibrinolytic-coagulation imbalance anchored by PAI-1, thrombin, and tPA co-elevation is the defining plasma proteomic feature of severe HCPS and a candidate severity biomarker [137,145]. Second, endothelial barrier fragility is substantiated at multiple molecular levels: proteomically active coagulation plasma, virus-induced VEGF secretion, VE-cadherin internalization, and disruption of adherens junction integrity converge on a common functional endpoint of increased microvascular permeability [140,143,146]. Third, immune effector remodeling, including elevated IL-1β, TNF-α, and IL-6, alongside interferon-driven defense protein expression, correlates with disease severity and represents both a host defense response and a driver of vascular injury [137,147]. Fourth, species-specific proteostatic context determines whether infection resolves as persistent reservoir-adapted replication or escalates to immunopathology. The concordant emergence of these four signatures across fundamentally distinct sample types, including plasma, cultured endothelial cells, and naturally infected reservoir systems, and their consistency with the broader transcriptomic literature support their biological robustness rather than suggesting a platform-specific artifact [136,148].

### 4.4. Current Gaps in Proteomics and Opportunities for Expansion

Despite this mechanistic coherence, proteomics remains substantially under-deployed relative to both the biology of hantavirus disease and the technical capacity now available. Cohort-scale deep plasma discovery using data-independent acquisition (DIA) LC-MS/MS, which is common in COVID-19 and influenza research, routinely resolving more than 1000–2000 unique proteins per sample, has not been widely applied to hantavirus clinical cohorts [149,150]. Systematic phosphoproteomics, which enables high-throughput mapping of functional phosphorylation sites across large sample sets without prohibitive measurement timelines, remains largely unexploited [151]. Unbiased secretome profiling of infected endothelial cells, an approach that has illuminated cytokine networks, extracellular matrix remodeling, and intercellular signaling during influenza and coronavirus infection, has not been extensively applied in hantavirus systems [152]. This methodological gap is particularly consequential because hantavirus pathogenesis involves extensive post-transcriptional and post-translational regulation: fibrinolysis, complement regulation, coagulation cascade activation, adherens junction remodeling, platelet-endothelial mediator exchange, and extracellular protease activity are all governed by post-translational modifications, including phosphorylation, ubiquitination, proteolytic cleavage, and glycosylation, that transcriptomics cannot resolve [136,153]. Therefore, existing findings do not lack rigor or information content; they are mechanistically illuminating and biologically consistent, but the field has applied a powerful methodology selectively, and one of the largest gains in understanding HCPS and HFRS pathogenesis now depends on closing that deployment gap.

### 4.5. Lipid Metabolism and Cholesterol Biosynthesis as Central Control Nodes

The lipid metabolism axis is among the best-characterized pathways in hantavirus metabolomics. Cholesterol biosynthesis is a major determinant of viral replication capacity and macrophage inflammatory activation [154]. At the core of this axis is the transcriptional program of SREBP2, a master regulator of cellular cholesterol homeostasis [155]. The upstream long non-coding RNA NEAT1 (notably, the NEAT1-2 isoform) potentiates SREBP2 activity by promoting Srebf2 expression and interacting with SREBP2. This results in enhanced inflammatory macrophage activation and limits viral spread. Importantly, NEAT1-2 expression in HFRS patients’ monocytes was negatively correlated with viral load and disease progression. This suggests that the lncRNA-sterol regulatory circuit functions as a mechanistic driver and potential biomarker for severity [156].

Orthogonally, interferon-stimulated production of 25-hydroxycholesterol (25HC) catalyzed by cholesterol 25-hydroxylase suppresses 3-hydroxy-3-methylglutaryl-CoA reductase (HMGCR) and restricts hantavirus replication through a mechanism that functionally overlaps with statin activity. The observation that pitavastatin, simvastatin, fluvastatin, and atorvastatin each recapitulate this inhibition in a dose-dependent manner suggests the pharmacological tractability of the cholesterol flux axis. The convergence of oxysterol signaling, SREBP2-driven transcriptional remodeling, and statin-sensitive HMGCR activity around a shared lipid biosynthetic program reveals a fundamental biological tension, and the same cholesterol flux that hantaviruses require for replication is simultaneously the target of the host’s primary antiviral lipid defense, a duality with direct implications for therapeutic design [157].

### 4.6. Energy Metabolism: Bioenergetic Divergence and Unresolved Intermediates

Energy metabolism represents a second, structurally important but less resolved axis in hantavirus pathogenesis. Species-divergent bioenergetic phenotypes have been documented in infected macrophages: RBP-J-deficient murine macrophages, reflecting a Notch-enforced transcriptional restraint characteristic of late-phase infection, maintain elevated extracellular acidification rates consistent with glycolytic dominance, while showing decreased oxygen consumption relative to wild-type controls at 36 h post-infection [131]. This bioenergetic divergence between murine and human macrophage responses is significant not merely as a species difference, but because it reveals the fundamental limitation of conventional metabolic readouts.

Transcriptomic profiling and extracellular flux assays can resolve the net direction of metabolic shift, glycolytic versus oxidatively dominant, but cannot identify the specific TCA cycle intermediates, lipid mediators, or intracellular redox couples that likely differentiate replication-permissive macrophage states from those executing immunopathogenic programs in human HFRS [158]. Mitochondrial metabolites, including citrate, succinate, and itaconate, play dual energetic and immunosignaling roles in inflammatory activation, and although pathway enrichment analyses implicate their involvement, hantavirus-specific, discovery-scale characterization of these intermediates in clinical specimens remains absent [158]. The gap between inferred metabolic phenotype and measured metabolite identity remains an important unresolved question in hantavirus immunometabolism.

### 4.7. One-Carbon Metabolism and Hematologic Disease Severity

A more diagnostically tractable strategy is illustrated by the intersection of one-carbon metabolism and hematologic disease severity. Circulating NRBCs, which are a quantifiable phenotype reflecting erythropoietic maturation arrest, are enriched in severe HFRS, and affected patients show depressed plasma vitamin B12 and folate concentrations [125]. One-carbon metabolism, dependent on both cofactors, is indispensable for DNA synthesis in rapidly proliferating hematopoietic precursors; its disruption lengthens S-phase, impairs erythroid differentiation, and drives megaloblastic precursors into circulation [159]. This triangulation, connecting a plasma metabolite signature (B12/folate deficiency) to a single-cell-defined blood phenotype (NRBC frequency) and to a clinical severity gradient, exemplifies a biomarker development paradigm that is mechanistically grounded, clinically actionable, and achievable without genome-scale cohort resources [125]. Its practical advantage over untargeted metabolomics lies precisely in this mechanistic parsimony: a focused panel of pathway intermediates, including homocysteine as a functional readout of one-carbon flux, could stratify disease severity and illuminate a discrete biological mechanism simultaneously [159,160].

### 4.8. Emerging Metabolic Dimensions and Uncharacterized Opportunities

Despite these advances, several metabolic dimensions remain largely uncharacterized and provide major opportunities in the field. Oxidized phospholipid mediators, containing specialized pro-resolving lipids and non-enzymatically oxidized polyunsaturated fatty acids, modulate macrophage activation and viral persistence in multiple infection models [161]. However, they have not been systematically profiled in hantavirus pathogenesis. Mitochondrial intermediates beyond the core TCA cycle, particularly fumarate and 2-hydroxyglutarate, engage epigenetic regulatory circuits and redox signaling [162]. These intermediates are mechanistically relevant to endothelial and immune dysfunction but lack hantavirus-specific investigation. Vascular permeability mediators and the metabolic sequelae of bradykinin-induced calcium dysregulation are central to hantavirus-induced capillary leak syndrome yet remain largely unexplored using metabolomic approaches [153]. Such approaches could capture organ-specific metabolic injury, predominantly renal injury in HFRS and pulmonary-endothelial injury in HCPS, without requiring tissue sampling [163]. Addressing these gaps through targeted-discovery hybrid approaches anchored to mechanistic hypotheses but powered by untargeted detection would substantially deepen the metabolic framework of hantavirus pathogenesis and identify therapeutic targets currently invisible to transcriptomic inference alone. To facilitate interpretation of the major proteomic and metabolomic findings discussed above, Table 2 summarizes the principal molecular signatures, their mechanistic roles in hantavirus pathogenesis, current biomarker potential, and opportunities for therapeutic intervention.

## 5. Integrative Multi-Omics and AI-Driven Frameworks for Hantavirus Surveillance and Therapeutic Discovery

**Figure 5:**
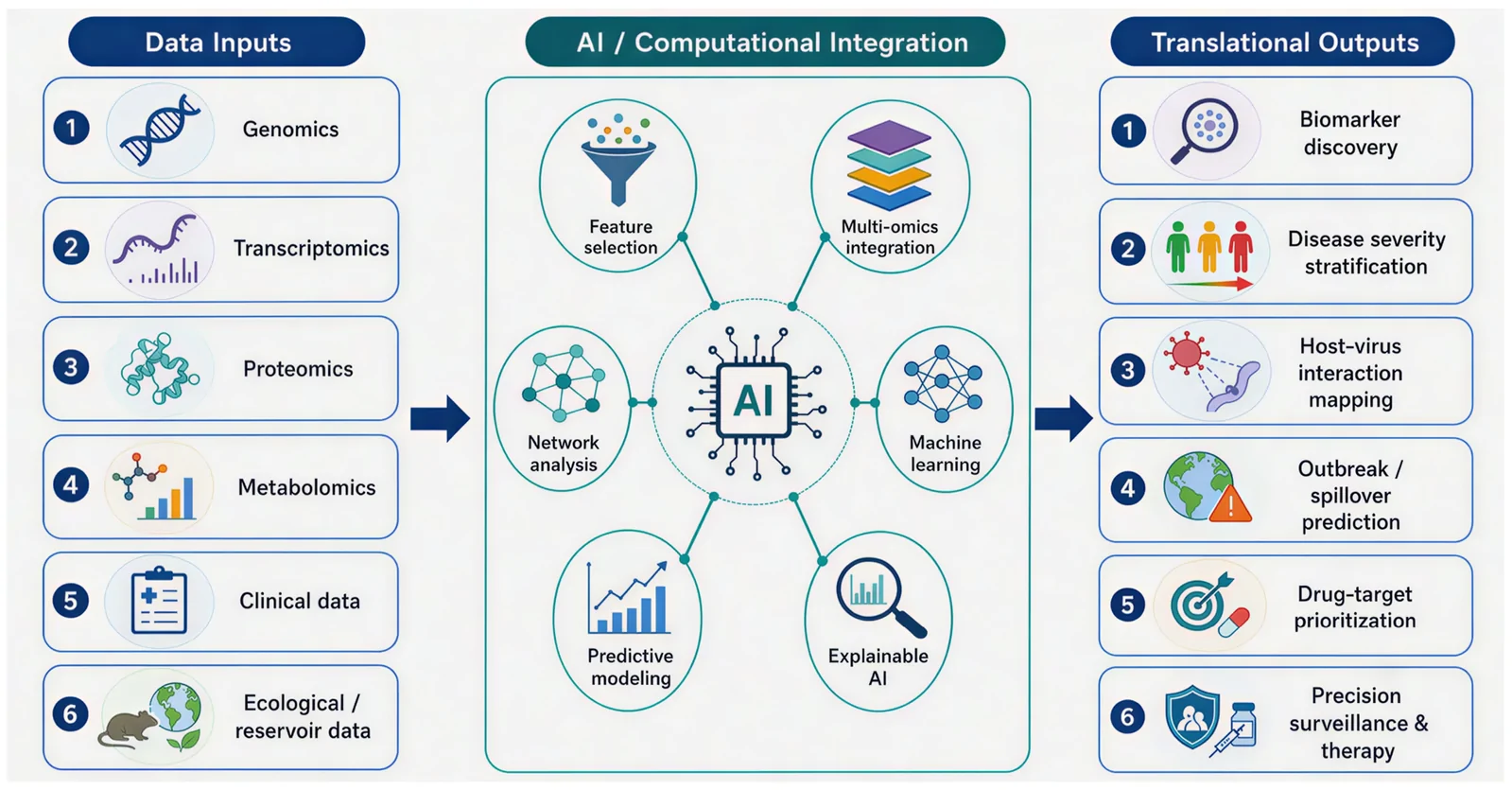
AI-enabled integration of multi-omics data in hantavirus research. Genomic, transcriptomic, proteomic, metabolomic, clinical, and ecological data are integrated through AI-based computational approaches to support biomarker discovery, disease stratification, outbreak prediction, drug-target prioritization, and precision surveillance and therapy. The image was created by the authors in the Canva online program (https://www.canva.com/, accessed on 29 June 2026).

The technical challenge of meaningful multi-omics integration lies not merely in data acquisition but rather in the conceptual and computational integration of these heterogeneous data types into coherent biological narratives. Several methodological frameworks have emerged for multi-omics integration, each with distinct strengths and limitations relevant to hantavirus research. A schematic overview of the proposed AI-enabled multi-omics integration framework is presented in Figure 5.

### 5.1. Multi-Omics Integration Strategies: Conceptual Frameworks and Methodological Approaches

Network-based integration approaches build biological interaction networks that include multiple omics layers as interconnected node types [172]. In this framework, genomic variants identified in a given hantavirus strain could be associated with alterations in host transcription factor binding sites, which are then associated with downstream alterations in host gene expression, which then map to alterations in protein abundance and post-translational modifications, and finally cascade into altered metabolite levels in infected cells [173]. Tools such as STRING (Search Tool for the Retrieval of Interacting Genes/Proteins) and OmniPath have been adapted in order to support multi-omics data by creating heterogeneous biological networks in which nodes correspond to genes, proteins, metabolites, and viral components, and edges encode regulatory, physical, and functional relationships between the different entities [174,175].

In hantavirus applications, incorporation of network-based information may reveal how specific features of the viral genome (e.g., specific mutations in the RNA-dependent RNA polymerase) spread in the host regulatory landscape to cause disease-relevant phenotypes [176]. For example, in a hypothetical integrative network analysis, a specific hantavirus Gn glycoprotein variant (genomic layer) might be found to have a preferential binding to β3 integrin variants encoded by specific host genetic variants (genomic layer), which induces the expression of the transcription factor STAT1 (transcriptomic layer), resulting in upregulation of interferon-stimulated genes and abundance of phosphorylated STAT1 protein (proteomic layer), which culminates in metabolic reprogramming towards glycolysis and lipid peroxidation (metabolomic layer) [158,177,178,179]. Such an integrated understanding directly implicates specific intervention points (e.g., STAT1 inhibitors, integrin antagonists), and predicts which patient populations might benefit most from such interventions based on their viral genotype and host genetic background [180]. Network integration-based methods are especially useful for identifying cross-layer regulatory dependencies and for detecting hub molecules (genes, proteins, or metabolites) that exert disproportionate influence across multiple biological layers and thus represent high-value therapeutic targets [181].

A complementary integration strategy employs dimensionality reduction techniques that can accommodate multiple omics modalities simultaneously. Methods such as multiple canonical correlation analysis (mCCA), multi-omics factor analysis (MOFA), and joint dimensionality reduction approaches (e.g., hierarchical stacked generalization) reduce each omics dataset to a lower-dimensional representation of latent biological factors, then identify concordances and correlations between these latent representations across omics layers [182,183]. MOFA and related approaches have proven particularly effective for disease subtyping and patient stratification in heterogeneous disease cohorts. The method constructs a matrix factorization model, where each row represents a sample (e.g., an infected patient or animal model), each column represents an omics feature (genes, proteins, metabolites), and the factorization yields a set of latent factors that explain variation across all omics layers. Samples that cluster together in latent factor space represent patients with similar integrated omics signatures, even if individual omics layers show significant heterogeneity [182]. For hantavirus research, MOFA applied to patient cohorts with varying disease severity could potentially identify previously unrecognized disease endotypes characterized by distinct integrative molecular signatures, for instance, a hyperinflammatory-metabolic-dysregulation endotype characterized by elevated interferon response (transcriptomic), enhanced phosphorylation of TLR-signaling proteins (proteomic), and glycolytic-to-lipolytic metabolic switching (metabolomic), versus a replication-permissive, immune-blunted endotype with suppressed interferon signaling and preserved oxidative phosphorylation.

In the context of viral infection, the kinetics of molecular events are often as informative as the steady-state molecular signatures. Hantavirus infection is characterized by distinct clinical phases (prodromal, hemorrhagic, and recovery), and the molecular landscape differs markedly between phases. Temporal integration methods construct data matrices in which rows are time points, and columns are omics features. This enables the identification of temporal regulatory patterns and causal chains of molecular events [184]. Gaussian process regression, dynamic causal modeling, and time-series factor analysis extend dimensionality reduction approaches to explicitly model temporal dynamics within the integrated multi-omics framework [185,186,187].

For hantavirus, longitudinal multi-omics sampling from patient cohorts at multiple disease stages (acute viremia, hemorrhagic phase, convalescence) could reveal the temporal sequence of molecular events driving disease progression. For example, an early transcriptomic signature of interferon-α induction might temporally precede the proteomic emergence of elevated phosphorylated NF-κB and TNF receptor-associated factors, which in turn temporally precede the metabolomic shift toward anaerobic glycolysis and lactate accumulation [188]. Such temporal integration explicitly reveals mechanistic causality and defines therapeutic windows for intervention.

A particularly promising integration strategy is to use existing biological knowledge bases and literature-derived interaction networks to constrain and guide the integration process. Rather than using computational algorithms to identify associations de novo (which is vulnerable to false-positive correlations in high-dimensional data), knowledge-guided integration uses prior information on known viral-host interactions, innate immune signaling pathways, metabolic dependencies, and evolutionary constraints to guide the integration toward biologically meaningful and interpretable solutions [189,190]. For hantavirus, knowledge-guided integration could incorporate prior evidence that hantavirus nucleocapsids interact with host proteins involved in innate immune recognition (e.g., RIG-I, MDA5) [191], that glycoproteins engage endothelial surface receptors (e.g., integrins, β3-integrins) [143], and that viral proteins activate NF-κB and interferon signaling [192]. These constraints ensure that inferred multi-omics associations are consistent with mechanistic understanding, rather than identifying statistically significant but biologically irrelevant correlations.

### 5.2. Multi-Omics Applications in Hantavirus Biomarker Discovery and Patient Stratification

An important but relatively under-investigated application of multi-omics integration in hantavirus research is the discovery of integrated biomarker panels for predicting disease severity, response to treatment, and clinical outcomes [193,194]. At present, clinical management of hantavirus infection is based mainly on clinical signs and laboratory parameters (hematocrit, platelet count, serum creatinine), which provide limited insight into disease mechanisms and are often observed relatively late in the course of disease [195,196]. Alternatively, integrated multi-omics biomarker signatures may allow early prediction of disease severity during the acute febrile phase, and prior to the development of hemorrhagic manifestations, and therefore direct intensive supportive care, antiviral therapy, and immunomodulatory interventions to high-risk patients [24].

Successful multi-omics disease subtyping has been demonstrated in COVID-19 research. Integrative analysis of genomic host variation, transcriptomic immune responses, proteomic inflammatory markers, and metabolomic signatures of metabolic dysregulation has identified distinct disease endotypes (hyperinflammatory, thrombotic-vasculopathic, immunosuppressed) that correspond to clinical outcomes and predict treatment response to targeted immunomodulatory therapies [197,198]. Similarly, in hantavirus research, hypothetical endotypes could be defined by similar approaches, such as the following categories.

Dysregulated endothelial-immune endotype: over-activation of the endothelial surface (upregulation of angiopoietin-2, VE-cadherin shedding, increased number of circulating endothelial cells in the proteomic layer) [140,168,199], persistent interferon-α signaling and aberrant TNF-α-driven inflammation (transcriptomic layer) [192,200], and metabolic reprogramming towards glycolytic-lactate accumulation (metabolomic layer) [201]. This endotype would predict severe vascular permeability, hemorrhagic manifestations, and possible response to immunomodulatory therapy targeting TNF-α or endothelial stabilization (e.g., angiopoietin-1 analogs).

Viral replication-permissive endotype: characterized by suppressed interferon-beta and lambda responses despite high viral replication (transcriptomic) [202], attenuated antigen-presentation signatures (proteomic) [203], and metabolic profiles consistent with maintenance of survival signals (metabolomic) [204]. This endotype would predict lower initial symptom severity but potential for delayed viral clearance and prolonged viremia, necessitating direct antiviral therapy.

Metabolic exhaustion endotype: characterized by profound metabolic dysregulation with depletion of energy substrates, upregulation of autophagy-related genes, and accumulation of potentially toxic lipid metabolites (metabolomic) [205,206], coincident with suppressed adaptive immune gene expression and cellular exhaustion markers (transcriptomic) [207], and reduced abundance of metabolic enzymes and mitochondrial proteins (proteomic). This endotype would predict a high risk of multiorgan failure despite moderate initial viremia, requiring aggressive metabolic support and antioxidant therapy.

Discovery of such integrative endotypes would require prospective, well-powered cohort studies with serial multi-omics sampling from hantavirus patients across the full spectrum of disease severity. However, given the rarity of severe hantavirus infections in individual centers, multicenter consortium approaches and biobank-derived retrospective studies of archival samples could be feasibly implemented [208]. The integration of multi-omics data is not only useful for biomarker discovery but also provides a system-level understanding of hantavirus pathogenesis.

Table 3 shows how omics layers can provide complementary biological information that can be integrated using AI-driven analytical frameworks to facilitate disease stratification, prognostic modelling, and future precision medicine approaches.

### 5.3. AI and Machine Learning Frameworks

Climate and ecological factors drive hantavirus outbreaks (e.g., rodent mast years). Machine learning can integrate time-series case data with weather/land-use to forecast risk. A binary support vector machine (SVM) classifier was developed for forecasting human PUUV outbreaks in Germany. The model used just three lagged weather features (soil temperature and sunshine) and a spatial synchrony transformation of incidence. The model attained 85% sensitivity and 71% precision in predicting high-incidence outbreak years. The investigators also introduced a PUUV Outbreak Index (POI) to quantify outbreak synchrony. Such models are simple yet effective and can serve as heuristic early warning tools [226]. More complex methods (random forests, LSTM/ARIMA hybrids) could address nonlinearity and temporal trends. Geospatial ecological models have been proposed to predict hantaviral spillover through the identification of reservoir habitats and key ecological drivers [227]. In practice, AI-enabled systems could pull real-time rodent population and climate data to alert when conditions favor an outbreak. HantaNet currently functions primarily as a hantavirus classification, genomic surveillance, epidemiological visualization, and standardized data-sharing platform rather than as a validated predictive outbreak-forecasting system [228]. Integrating externally validated AI risk forecasts into such platforms could represent a future development.

Learning host–pathogen networks from omics data is difficult. Machine learning methods can predict viral-host protein–protein interactions (PPIs) from sequence features or from similarity to known virus–host networks [229]. None of the ML PPI models published are specific for hantaviruses, but general approaches (e.g., embedding proteins in a vector space and classifying interactions) could be applied if interaction data are available. Multi-omics can be used to rank key host genes using graph-based methods (network propagation, graph neural networks). For example, shotgun approaches such as Lasso regression on host transcriptomics can identify master regulators whose perturbation predicts infection outcome. A recent network-based analysis prioritized the regulatory hubs by applying degree, betweenness, and closeness centrality measures on a PPI network obtained from the STRING database using transcriptomic data of HUVEC cells infected with HTNV. The most central nodes identified were ISG15, IRF1, CXCL10, STAT1, and DDX58. Conceptually, the hub-prioritization approach is similar to feature ranking in machine learning. While IRF7 and NF-κB-related signaling are part of the interferon/inflammatory response, they were not reported as top centrality hubs [230]. Further work could utilize causal inference or Bayesian networks to expand this framework and investigate regulatory pathways such as IL-6-associated inflammatory signaling leading to endothelial permeability.

AI can mine multi-omic signatures for diagnostic and prognostic markers. For example, multi-view ML (e.g., DIABLO, multi-block PLS) could identify mRNA-protein-metabolite signatures that can distinguish severe from mild HCPS [231]. Unsupervised learning (clustering, dimensionality reduction) applied to scRNA-seq of patient peripheral blood mononuclear cells can define immune cell states predictive of outcomes. Network embedding or autoencoder models may be used to integrate heterogeneous omics to identify biomarkers that are not apparent in single datasets. However, the small size of the datasets (often <100 patients) limits the model complexity, requiring careful cross-validation and feature regularization.

AI accelerates antiviral discovery by filtering vast chemical space. Structure-based ML (e.g., deep docking) can screen millions of compounds against viral targets (NP, Gn/Gc). A 2023 study used 2D-fingerprint virtual screening using favipiravir as a reference and docking/molecular dynamics (MD) to nominate Gn inhibitors [232]. More advanced approaches (graph neural networks predicting binding affinity) could further speed lead discovery. AI also assists drug repurposing: for instance, latent-space similarity or network proximity to known antivirals. A 2026 screen combined phenotypic screening with clustering of hit compounds (HSP90 inhibitors, mTOR inhibitors); ML could have optimized the selection of chemical libraries [233]. In drug-discovery studies, ML has predicted drug-virus activities (ChemProp, random forests on descriptors) [234] and integrating multi-omic host-response signatures (e.g., transcriptome of infection) into drug-disease graph models is another promising AI-driven strategy.

## 6. Conclusions

Hantaviruses remain a persistent and unpredictable global health threat for which no broadly approved antiviral or vaccine yet exists, and the central obstacle to progress is no longer a scarcity of molecular data but the absence of a framework that unifies it. This review has shown that each omics layer, examined in isolation, has already reshaped our understanding of hantavirus biology. Genomic surveillance has revealed a virus held under stringent negative selection, where point mutation is constrained, and reassortment serves as a major, ecologically contingent engine of genetic novelty. Transcriptomics has moved beyond bulk averages to expose disease-relevant cellular states, from infected immune and hematopoietic cell populations to the species-specific noncoding RNA circuits that may contribute to reservoir host tolerance of persistent infection while humans progress to acute disease. Proteomic and metabolomic studies, though comparatively underdeveloped, have converged on mechanistically robust signatures: a fibrinolytic-coagulation imbalance anchored by PAI-1, thrombin, and tPA; endothelial barrier failure; and cholesterol-biosynthetic and one-carbon metabolic nodes that link viral replication to disease severity.

The recurring lesson across these domains is that hantavirus pathogenesis is fundamentally systems-level and involves extensive post-transcriptional regulation, and therefore cannot be resolved by any single modality. Tropism, virulence, and clinical heterogeneity emerge from the simultaneous interplay of viral genotype, host receptor genetics, transcriptional rewiring, post-translational modification, and metabolic flux. Realizing the promise of integration will require deliberate methodological investment: network-based and dimensionality-reduction frameworks such as MOFA, temporal models that capture phase-resolved disease dynamics, and knowledge-guided strategies that constrain inference to biologically plausible relationships. Coupling these with AI and machine learning offers a credible path toward integrated biomarker panels, mechanistically defined disease endotypes for patient stratification, ecological forecasting of outbreaks, and accelerated, structure-guided antiviral and drug-repurposing pipelines targeting the polymerase and glycoprotein machinery.

Yet critical gaps persist. Spatial transcriptomics, deep cohort-scale plasma proteomics, and discovery-scale metabolomics remain largely unexploited; wildlife reservoir sampling is sparse and lacks longitudinal coverage, and multicenter cohorts with serial multi-omics sampling are still lacking. Closing these gaps will demand coordinated global data sharing, standardized analytical pipelines, and biobank-enabled consortium studies that overcome the rarity of severe cases. By transitioning from compartmentalized, reductionist inquiry toward a genuinely integrative, AI-enabled systems framework, the field can convert mechanistic insight into precision diagnostics, validated prognostic biomarkers, and rationally designed therapeutics, ultimately improving patient outcomes and strengthening preparedness against this evolving threat. From the authors’ perspective, current omics research has markedly deepened our understanding of hantavirus biology, yet the field remains methodologically fragmented, with most investigations focusing on single-layer datasets rather than truly integrated multi-omics frameworks. This fragmentation limits the reconstruction of the complex molecular networks that govern viral pathogenesis and host immune responses. In addition, heterogeneous experimental designs and analytical pipelines across studies reduce comparability and reproducibility. Moving forward, progress will depend on standardized multi-omics workflows, longitudinal sampling strategies, and the incorporation of AI-enabled integrative analyses to identify reliable biomarkers, elucidate systems-level mechanisms, and accelerate the translation of omics insights into clinical practice.

## Figures and Tables

**Table 1:** Comparative genomic organization of representative hantavirus species, including segment lengths and encoded proteins.

Species	S Segment Length (kb)/Encoded Proteins	M Segment Length (kb)/Encoded Proteins	L Segment Length (kb)/Encoded Proteins	Ref
*Orthohantavirus hantanense*	1.696/N protein (429 aa)	3.616/GPC (1135 aa)	6.530/RdRp (2150 aa)	[78,79,80]
*Orthohantavirus seoulense*	1.771/N protein (429 aa)	3.651/GPC (1133 aa)	6.530/RdRp (2151 aa)	[81,82,83]
*Orthohantavirus puumalaense*	1.830/N protein (433 aa) & NSs (90 aa)	3.682/GPC (1148 aa)	6.530/RdRp (2156 aa)	[84,85,86]
*Orthohantavirus sinnombreense*	2.059/N protein (428 aa) & putative NSs (63 aa)	3.696/GPC (1140 aa)	6.562/RdRp (2153 aa)	[87,88,89,90]
*Orthohantavirus andesense*	1.871/N protein (428 aa) & NSs (63 aa)	3.671/GPC (1138 aa)	6.562/RdRp (2152 aa)	[91,92,93]

Abbreviations: aa, amino acid; GPC, glycoprotein precursor; kb, kilobase; L, large genomic RNA segment; M, medium genomic RNA segment; N protein, nucleocapsid protein; NSs, nonstructural protein; RdRp, RNA-dependent RNA polymerase; S, small genomic RNA segment.

**Table 2:** Proteomic and metabolomic hallmarks of hantavirus pathogenesis: mechanistic insights, biomarkers, and translational opportunities.

Molecular Signature	Omics Domain	Mechanistic Role	Association with Disease Severity	Biomarker Potential	Therapeutic Opportunity	Ref
PAI-1 ↑	Proteomics	Inhibition of fibrinolysis	Severe HCPS	High	Anti-fibrinolytic modulation	[145,164]
Thrombin ↑	Proteomics	Coagulation activation	Severe vascular injury	High	Coagulation-targeted therapies	[137,165]
tPA Dysregulation	Proteomics	Fibrinolytic imbalance	Pulmonary edema and leakage	High	Endothelial stabilization	[137,166]
VEGF ↑	Proteomics	Endothelial permeability	Capillary leakage	Moderate-High	Anti-VEGF strategies	[148,167]
Angiopoietin-2 ↑	Proteomics	Endothelial activation	Severe disease progression	High	Vascular protection	[145,168]
IL-6 Signaling ↑	Proteomics	Junction disruption	Increased permeability	Moderate	Cytokine-targeted therapy	[136,139]
NEAT1-SREBP2 Axis	Metabolomics/Transcriptomics	Cholesterol regulation	Lower viral burden when activated	High	Host-directed therapy	[156,169]
25-Hydroxycholesterol ↑	Metabolomics	Antiviral lipid mediator	Restriction of replication	High	Oxysterol-based interventions	[157,170]
HMGCR Pathway	Metabolomics	Cholesterol synthesis	Supports viral replication	Moderate	Statins	[157]
Glycolytic Shift	Metabolomics	Immunometabolic activation	Inflammatory disease phenotype	Moderate	Metabolic reprogramming	[158,171]

**Note:** ↑ indicates increased expression or elevated levels.

**Table 3:** Integrated multi-omics signatures, biological mechanisms, and AI-enabled translational applications in hantavirus infection.

Omics and Systems Biology Layer	Representative Molecular Signatures	Biological Process/Pathway	Clinical Relevance	AI-Enabled Application	Translational Potential	Ref
Viral Genomics	Viral mutations, segment reassortment, and sequence variation in S, M, and L segments	Viral adaptation, host specificity, transmissibility, and immune escape	Identification of virulence-associated variants and outbreak surveillance	Genomic classification, phylogenetic prediction, outbreak forecasting	Precision surveillance and epidemiological risk assessment	[209,210]
Host Genomics	HLA variants, immune-regulatory polymorphisms, susceptibility-associated loci	Host genetic susceptibility and immune responsiveness	Prediction of disease severity and individual risk profiles	Genetic risk modeling and patient stratification	Personalized risk assessment	[211,212]
Transcriptomics	ISG15, IFIT1, MX1, OAS1, STAT1, IRF7, CXCL10 expression signatures	Interferon signaling, antiviral response, cytokine activation	Early detection of severe immune dysregulation	Gene-expression classifiers and severity prediction models	Early diagnosis and prognostic biomarker panels	[213,214]
Proteomics	Cytokines, chemokines, angiopoietin-2, VEGF, endothelial injury markers	Endothelial dysfunction, vascular leakage, and inflammation	Prediction of hemorrhagic complications and capillary leak syndrome	Multi-protein predictive models and feature selection algorithms	Clinical severity monitoring and therapeutic targeting	[214,215]
Metabolomics	Altered lipid metabolism, amino-acid dysregulation, oxidative stress metabolites, and energy metabolism markers	Cellular stress response, mitochondrial dysfunction, immunometabolic reprogramming	Identification of metabolic signatures associated with disease progression	Metabolic pattern recognition and outcome prediction	Precision metabolic monitoring and treatment optimization	[216,217]
Integrated Multi-Omics Networks	Cross-platform gene–protein–metabolite interaction signatures	Systems-level host–virus interaction networks	Identification of robust composite biomarkers	Network analysis, graph-based learning, multi-modal AI models	High-confidence biomarker discovery	[218,219]
Reservoir Host Omics	Immune-tolerance markers, PD-L1 signaling, host adaptation signatures	Viral persistence without overt disease	Understanding mechanisms of reservoir competence and spillover	Comparative machine-learning models across species	Spillover prediction and zoonotic risk assessment	[203,220]
Longitudinal Multi-Omics Profiling	Dynamic molecular signatures during the acute, peak, and recovery phases	Disease evolution and recovery trajectories	Time-dependent prediction of disease progression	Temporal machine learning and trajectory modeling	Dynamic patient monitoring and precision intervention	[188,221,222]
Explainable AI-Derived Biomarker Panels	Multi-layer molecular signatures selected through interpretable algorithms	Integrated disease pathways	Clinically actionable decision-support biomarkers	Explainable AI (XAI), SHAP-based models, interpretable classifiers	Regulatory acceptance and clinical implementation	[223,224]
Precision Medicine Framework	Integrated genomic, transcriptomic, proteomic, metabolomic, and clinical data	Comprehensive disease mechanisms	Patient stratification and individualized management	Multi-modal predictive platforms and digital decision-support systems	Future precision medicine ecosystem for hantavirus infection	[224,225]

## Data Availability

No new data were created or analyzed in this study. Data sharing is not applicable to this article.

## Abbreviations

The following abbreviations are used in this manuscript:

ARIMA	Autoregressive integrated moving average
B2M	Beta-2 microglobulin
BSL-2	Biosafety level 2
Cas13	CRISPR-associated protein 13
COVID-19	Coronavirus disease 2019
CRISPR	Clustered regularly interspaced short palindromic repeats
DAF	Decay-accelerating factor
DIA	Data-independent acquisition
DIABLO	Data Integration Analysis for Biomarker discovery using Latent cOmponents
dN/dS	Ratio of nonsynonymous to synonymous substitution rates
Gc	Glycoprotein C
Gn	Glycoprotein N
GPC	Glycoprotein precursor
GTP	Guanosine triphosphate
HCPS	Hantavirus cardiopulmonary syndrome
HFRS	Hemorrhagic fever with renal syndrome
HLA	Human Leukocyte Antigen
HMGCR	3-hydroxy-3-methylglutaryl-CoA reductase
HPS	Hantavirus pulmonary syndrome
HSP90	Heat shock protein 90
HTNV	Hantaan virus
HUVEC	Human umbilical vein endothelial cell
IFN	Interferon
IL-1β	Interleukin-1 beta
IL-6	Interleukin-6
ISG	Interferon-stimulated gene
JAK-STAT	Janus kinase-signal transducer and activator of transcription
kb	Kilobase
L	Large genome segment
LC-MS/MS	Liquid chromatography-tandem mass spectrometry
LDG	Low-density granulocyte
L-M	Large–medium segment reassortment
lncRNA	Long non-coding RNA
LNP	Lipid nanoparticle
L-S	Large-small segment reassortment
LSTM	Long short-term memory
MAVS	Mitochondrial antiviral-signaling protein
mCCA	Multiple canonical correlation analysis
MDA5	Melanoma differentiation-associated protein 5
MD	Molecular dynamics
MHC	Major histocompatibility complex
miRNA	MicroRNA
ML	Machine learning
MOFA	Multi-omics factor analysis
mRNA-LNP	Messenger RNA-lipid nanoparticle
M-S	Medium–small segment reassortment
mTOR	Mechanistic target of rapamycin
N	Nucleocapsid protein
NEAT1	Nuclear paraspeckle assembly transcript 1
NF-κB	Nuclear factor kappa B
NP	Nucleocapsid protein
NRBC	Nucleated red blood cell
NSs	Non-structural protein
PAI-1	Plasminogen activator inhibitor-1
PBMC	Peripheral blood mononuclear cell
PCDH1	Protocadherin-1
PCR	Polymerase chain reaction
PD-L1	Programmed death-ligand 1
PHV	Prospect Hill virus
PLS	Partial least squares
PMN	Polymorphonuclear neutrophil
POI	PUUV Outbreak Index
PPI	Protein–protein interaction
PRIDE	Proteomics Identifications Database
PUUV	Puumala virus
RBP-J	Recombination signal binding protein for immunoglobulin kappa J region
RdRp	RNA-dependent RNA polymerase
RIG-I	Retinoic acid-inducible gene I
T-qPCR	Reverse-transcription quantitative polymerase chain reaction
scRNA-seq	Single-cell RNA sequencing
SREBP2	Sterol regulatory element-binding protein 2
STAT1	Signal Transducer and Activator of Transcription 1
STRING	Search Tool for the Retrieval of Interacting Genes/Proteins
SVM	Support vector machine
TCA	Tricarboxylic acid
TFH	T follicular helper cell
TLR	Toll-like receptor
TNF	Tumor necrosis factor
tPA	Tissue plasminogen activator
TULV	Tula virus
uPA	Urokinase plasminogen activator
UTR	Untranslated region
VE-cadherin	Vascular endothelial cadherin
VEGF	Vascular endothelial growth factor
VEGFR2	Vascular endothelial growth factor receptor 2
XAI	Explainable artificial intelligence
