# Correction: Najafi et al. Omics Approaches in Hantavirus Research: Current Advances, Challenges, and Future Perspectives. *BioTech* 2026, *15*, 58

**DOI:** 10.3390/biotech15040085

**Published:** 2026-09-30

**Authors:** Soroosh Najafi, Maryam Jojani, Kianoosh Najafi, Giovanni N. Roviello

**Affiliations:** 1Scientific and Production Center “Armbiotechnology” NAS RA, 14 Gyurjyan Str., Yerevan 0056, Armenia; najafi1380@gmail.com; 2School of Medicine and Surgery, University of Naples ‘Federico II’, Via S. Pansini 5, I-80131 Naples, Italy; aidajojani@gmail.com (M.J.); najafi2725@gmail.com (K.N.); 3Institute of Biostructures and Bioimaging (IBB), CNR National Research Council of Italy, 80145 Naples, Italy

In the original publication [1], two errors were identified in the main text, and one reference required replacement with a more appropriate citation, as detailed below.

## Text Corrections


In section 5.3, the word “can” in the phrase “ML (e.g., DIABLO, multi-block PLS) can identify mRNA-protein-metabolite signature” should be replaced with “**could**”.In section 5.3, the words “influenza/COVID” should be replaced with “**drug-discovery**”.


## References

Replace the original Reference [167] with a new reference:

Reference currently published:
167.Martinelli, E.; Tharinger, H.; Frank, I.; Arthos, J.; Piatak, M., Jr.; Lifson, J.D.; Blanchard, J.; Gettie, A.; Robbiani, M. HSV-2 infection of dendritic cells amplifies a highly susceptible HIV-1 cell target. *PLoS Pathog.* **2011**, *7*, e1002109.

Reference proposed by the authors:

Gavrilovskaya I, Gorbunova E, Koster F, Mackow E. Elevated VEGF Levels in Pulmonary Edema Fluid and PBMCs from Patients with Acute Hantavirus Pulmonary Syndrome. Adv Virol. 2012;2012:674360. doi: 10.1155/2012/674360. Epub 2012 Aug 22. PMID: 22956954; PMCID: PMC3432326.

The citation to reference [167] is present in Table 2 in the main text, but it will not be affected. With this correction, the order of the references will not be changed.

The authors apologize for this oversight and confirm that the scientific conclusions of the article remain unaffected.

This correction was approved by the Academic Editor. The original publication has also been updated.
